# Effect of Rural-Urban Migration on Age at Marriage Among Adolescent Girls in Bangladesh

**DOI:** 10.3389/fpubh.2022.840145

**Published:** 2022-07-06

**Authors:** Jannatul Ferdous Antu, Kausar Parvin, Hasan Mahmud Sujan, Mahfuz Al Mamun, Ruchira Tabassum Naved

**Affiliations:** Health Systems and Population Studies Division, International Centre for Diarrhoeal Disease Research Bangladesh (icddrb), Dhaka, Bangladesh

**Keywords:** age at marriage, child marriage, rural-urban migration, propensity score matching (PSM), adolescent girls, Bangladesh

## Abstract

**Objectives:**

Bangladesh reports one of the highest rates of child marriage (CM) (59%) in the world and the highest rate within South Asia. Age at marriage of girls is a critical human rights and developmental issue in Bangladesh. Migration has been documented to be positively associated with age at marriage. Bangladesh experiences one of the highest rates of rural to urban migration in the world. An increase in rural-urban migration of adolescent girls has been observed over the last few decades in Bangladesh with the expansion of employment opportunities particularly in the ready-made garment industry (RMG). This analysis explores the effect of migration on age at marriage and CM among adolescent girls living in urban slums of Dhaka.

**Methods:**

The sample was selected from icddr,b's Urban Health and Demographic Surveillance System (UHDSS) and comprises of never-married adolescent girls aged 15–19, who migrated in from rural Bangladesh to slums in and around Dhaka during 2015–2019. These in-migrants were matched with their rural counterparts from icddr,b's Matlab HDSS (MHDSS), using one to one nearest neighbor matching with caliper 0.1 using propensity score matching (PSM) method. The sample derived included a total of 2,700 never-married adolescent girls from Dhaka and Matlab. The association between migration and age at first marriage was estimated using a linear regression model and the effect of migration on CM was explored using logistic regression analyses.

**Results:**

The in-migrants perfectly matched with their rural peers in terms of age, household wealth and religion. However, their income earning status was hugely different. Urban migrants had significantly higher age at marriage than the rural non-migrants for both 15–19 (Coefficient, 1.77; 95% CI, 1.07–2.46) and 20–24 years age group (Coefficient, 2.87; 95% CI, 2.18–3.55). The migrant girls aged 20–24 years were 71% (aOR, 0.29; 95% CI, 0.12–0.69) less likely to get married before CM age bar in Bangladesh compared to their rural counterparts.

**Conclusion:**

Migration has a positive effect on delaying marriage and reducing CM among adolescent girls. Findings from this study suggest that CM can be reduced by creating educational and economic opportunities for females.

## Introduction

Worldwide, child marriage (CM) is a major issue related to denial of child rights, negatively contributing to public health and hindering individual and national development. Globally, more than 600 million girls are married before they reach 18 years (1). Moreover, around 12% are married before 15 years of age (2). Around half of the global burden of CM is contributed by South Asia (1). CM has adverse physical and mental health outcomes for the child bride, as well as for her offspring. Child brides are more likely to suffer from malnutrition, unintended pregnancy, pregnancy related complications and death (3–6). Moreover, a child born to a child bride is at higher risk of dying within their first year of life (7). CM increases the risks of anxiety, depression and suicidal attempt for young girls (8, 9). It also limits the opportunities for education and employment for girls (10) and increases the likelihood of experiencing intimate partner violence (11).

Bangladesh reports one of the highest rates of CM (59%) in the world and the highest in South Asia (12). There are rural urban differences in the rate of CM, with consistently higher rates being reported by rural Bangladesh (61% in rural vs. 55% in urban areas) (13). Thus, the median age at first marriage in rural areas is 16 years and urban areas is 17 (BDHS 2017–18). However, the median age at first marriage remains low in urban slums (14). The literature shows that poverty, pervasive patriarchal social norms, dowry system, concerns about family reputation are some important risk factors of CM (15); while education, higher wealth status, urban residence and income earning are protective factors against CM in Bangladesh (16, 17).

A link between female rural-urban migration and delayed age at marriage has been suggested in the literature (14, 18). Previous literature suggests that migrants and those who earn an income were able to delay their marriage. Another study has shown a significant positive association between a girl's exposure to readymade- garment industry (due to proximity of residence) and age at marriage (19). As many garment factories are located in and around Dhaka city this may influence age at marriage of the girls, who migrate in from rural areas. Female rural-urban migration has not only provided employment opportunities, but also has broadened the opportunity for education and development of livelihood skills. It also has a positive impact on social and economic empowerment for girls and women (20).

Bangladesh experiences one of the highest rates of rural-urban migration in the world. The number of migrants has increased significantly over the decades (21–23). Poverty, lack of economic opportunities, natural disasters are often cited as push factors for rural to urban migration (24). Historically, rural-urban migration in Bangladesh was dominated by men (25, 26). Previously, reasons for migration were very distinct for males and females. Thus, males mostly migrated to urban areas for employment, while only some females migrated to join their families living in urban areas (17). Over time, an increasing proportion of females started to migrate in search of employment (17). The rapid expansion of the garment industry and its demand for female laborers and other employment opportunities in Dhaka is a strong pull factor for female rural-urban migration. By 2011, rural-urban migration to Dhaka became dominated by females (27). An increase in rural-urban migration of unmarried adolescent girls is also attributed to the expansion of employment opportunities in the ready-made garment industry. This seems to be true judging by the fact that in 2018, 36% of never married adolescent girls aged 15–19 years, living in the slums, migrated to look for a job (Unpublished UHDSS data).

Studies on migration and age at marriage are mostly cross-sectional. Many of them are qualitative in nature or lack an appropriate comparison group (14, 18, 28). Thus, drawing a firm conclusion regarding the effect of migration on marriage based on these results has not been feasible. This paper aims to address this gap by comparing age at marriage in rural-urban migrants and non-migrants adolescent girls. The comparison group was selected applying propensity score matching to data from two novel Health and Demographic Surveillance Systems of icddr,b—UHDSS and MHDSS. The sample included adolescent girls, who migrated during 2015–2019.

## Method

### Study Setting and Study Design

This study used secondary data collected routinely as part of icddr,b's Health and Demographic Surveillance System (HDSS) in urban slums in and around Dhaka city and Matlab, a predominantly rural sub-district in Chandpur district of Bangladesh.

### Matlab HDSS

Matlab HDSS (MHDSS), the largest and oldest HDSS in the developing world was established in 1966. Since then, MHDSS is maintaining surveillance and collecting periodic census data. While MHDSS has recorded each birth, death and migration of all individuals in Matlab since 1966, collection of data on marriage and divorce started since 1975. In the MHDSS treatment area, additional data collection was introduced on maternal and child health services, immunization and, family planning services in 1978, and MHDSS data collection transitioned from monthly to bi-monthly since 2007. Trained female community health research workers collect the surveillance data. The mode of data collection shifted from pen and paper to a computerized database in 2007 (29). In addition to routine data collection periodic censuses were conducted in Matlab in the years 1966, 1974, 1982, 1996, 2005, and 2014 to collect socio-economic data (e.g., education, occupation, NGO membership, income sources, food security) (29). According to the 2014 census, MHDSS area includes 142 villages and 230,185 population.

### Urban HDSS

The Urban HDSS (UHDSS) was established in 2015, is the first HDSS in urban areas in Bangladesh. It comprises of two slums from Dhaka North–Korail and Mirpur; two slums from Dhaka South–Dhalpur and Shyampur; and Tongi from Gazipur city corporations. A baseline demographic and socio-economic census was conducted between September 2015 and January 2016 as the first step toward developing the UHDSS. This survey collected data on household wealth, family size, place of origin, cause of migration, duration of migration, dependency ratio, education of the girls, occupation, etc. The surveillance system with a prospective study design collects information on pregnancy outcome, death, marriage and divorce, change of household head, in and out migration, internal movement etc. every 3 months.

### Study Sample

This analysis included never-married girls, who were aged between 15 and 19 years at migration during 2015–2019. From the UHDSS, girls were selected using the following criteria: (1) migrated from rural areas between 2015 and 2019; (2) never-married at the time of migration; (3) currently living in the UHDSS area. From MHDSS, a matched sample of never-married adolescent girls aged 15–19 years between 2015 and 2019 were included in the analysis. For the purpose of this paper, the sample selected from UHDSS will be termed as migrants, and a set of matched sample from MHDSS as rural non-migrants (the comparison group). The samples were matched based on their age, religion and socio-economic status (SES). All the girls' data were followed from the base year 2015–2019 and their information were included in the analyses.

### Matching

Arbitrary control selection in research may result in a sample fraught with selection bias. To minimize selection bias in our sample, we matched the rural non-migrant sample with the urban migrant sample using the propensity score matching (PSM) technique (30). This allows us to reduce the difference in background characteristics between the two groups and minimize any potential selection bias. The propensity score is virtually a balancing score that confirms the distribution of variables which influence the exposure variables are same for case and comparison groups.

A matched set of observations were selected from both case (migrant urban sample) and comparison groups (non-migrant rural sample) based on propensity score's similarity, using logistic regression analysis (30). For selection of matched pairs we used the nearest neighbor matching method with a fixed caliper (31). A restriction of maximum acceptance distance was imposed on the propensity score, which is called caliper. In nearest neighbor matching with a fixed caliper method for a case subject, all available comparison subjects are identified on that imposed range of propensity scores and selects the nearest one. If more than one control subjects are available with the closest distance, then one comparison subject is chosen randomly for those subjects. If no comparison subject is found in the acceptable range, then the case subject would not match any comparison subject and be excluded from the matched sample frame (32).

There are no hard and fast rules for maximum acceptance of caliper and, researchers use a wide range of calipers (33, 34). Some studies suggest using caliper 0.2 for lower mean square error (MSE) (35). In this study, we set the caliper to 0.1 to assure higher similarities on matching variables. Implementing one to one nearest neighbor matching with caliper 0.1 yielded a total of 2,700 perfect/complete-matched adolescent girls aged 15–19 with 1,350 individuals per site. Considering age at first marriage as the outcome variable, 95% of confidence interval and 5% margin of error, the sample size were 105 for each site. We have considered mean age at first marriage as 19 and standard deviation as 3.5 at 2017 from MHDSS to calculate this sample size. This suggests that used sample size is quite large compared to the requirement.

As our sample included never married adolescent girls aged 15–19 migrated during last 5 years (2015–2019) found in the database in December, 2019, the age of the females in the sample ranged between 15 and 24. For this analysis, the sample was split into age groups, 15–19 and 20–24. In each HDSS site, 848 sample females were aged 15–19, and 502 were aged 20–24 years.

## Measures

### Outcome Variables

To assess the effect of rural-urban migration on age at marriage, our main outcome variable of interest was age at first marriage. Our secondary focus was to estimate the effect of rural-urban migration on proportion of ever-married females and child marriage. Proportion of ever-married was estimated using current marital status. The question on marital status included five response options: never married, currently married, divorced, separated and widowed. We re-categorized the codes into never-married (coded as “0”) and ever-married (coded as “1”).

Age at *first* marriage was used as a continuous variable.

In line with the definition of child marriage in Bangladesh, females married under the age of 18 were considered as child brides. The variable “child marriage” was constructed for women aged 20–24 years using information on age at first marriage and it was coded “1 = yes,” if age at marriage was <18, and “0 = no” otherwise.

### Exposure Variables

Migration was our main exposure variable with two categories, “1 = yes” if the participant was from the UHDSS who migrated from a rural area of Bangladesh and “0 = no” if the participant was a non-migrant from MHDSS.

### Matching Variables

Current age, religion and household wealth index were the three matching variables we used to select a matched pair of samples from UHDSS and MHDSS.

Age of the individual at December 2019 was considered as current age and used as a continuous variable the age range of the sample was between 15 and 24 years.

Religion of the study participants included two categories- Muslim (coded “0”) and Non-Muslim (coded “1”).

Household wealth index was calculated based on ownership of household assets common in both urban and Matlab HDSS data following similar methods used in UHDSS using principal component analysis (PCA). The scores yielded were then divided into quintiles and coded as, poorest = 1, poorer = 2, middle = 3, rich = 4, and richest = 5.

### Covariate

We used the income earning status of the study participants as a potential covariate and included in the models. The variable was “earns an income” coded as “1 = yes” and coded as “0 = no”.

### Statistical Analyses

Descriptive analysis was performed to describe the background characteristics of the study participants. Chi-square tests were performed to test whether the rural and urban samples were balanced in terms of matching variables. Average treatment effect (ATE) of migration was measured using linear regression model on the continuous outcome age at marriage. Logistic regression analyses were performed to measure the effect of rural-urban migration on the discrete outcome variables, i.e., (i) marital status and (ii) child marriage. Since the temporality exists between migration and the outcome variables, the effect we find could be interpreted as causal effect. This is possible both for binary outcome variables using logistic regression (30, 34, 35), and for continuous outcomes variables using linear regression analysis (30). While all the analyses were performed separately for age groups 15–19 and 20–24, the analyses related to child marriage were performed only on the 20–24 year olds. The level of significance was set at 5%.

All the regression analyses were adjusted for matching variables—age, religion and household wealth index, and the covariate—earns an income.

All the analyses were performed using Stata version 15 and RStudio version 1.3.1056. To perform the propensity score matching MatchIT package in RStudio and PSMATCH2 in Stata were used (36).

## Results

### Background Characteristics of the Study Sample

Table 1 shows the background characteristics of study sample by study groups (migrant and rural non-migrant) presented separately for age groups 15–19 and 20–24 years. There was no significant difference in age between the migrant and non-migrant adolescent girls for both age groups. In both age groups, proportions of ever-married adolescents at 2019 were significantly lower among the migrants (11% in 15–19 and 23% in 20–24 years age group) compared to their non-migrant rural counterparts (25% in 15–19 and 61% in 20–24 years age group). Huge differences existed in income earning status between the urban migrants and rural adolescent girls. Significantly more adolescents and young women were engaged in income-earning activities among the urban migrants (56% in 15–19 and 71% in 20–24 years age group) compared to the rural non-migrants (1% in 15–19 and 2% in 20–24 years age group).

**Table 1:** Background characteristics of propensity score matched sample by age group.

	**Age 15–19**			**Age 20–24**		
**Characteristics**	**Rural control**	**Rural to urban slum migrant**	***P*-value**	**Rural control**	**Rural to urban slum migrant**	***P*-value**
	***N* = 848**	***N* = 848**		***N* = 502**	***N* = 502**	
**Age**						
Mean age, year (range, SD)	17.7 (16–19, 1.04)	17.7 (15–19, 1.05)	0.944	20.9 (20–24, 0.89)	20.9 (20–23, 0.88)	0.887
**Household wealth, % (** ***n*** **)**						
Poorest	84.9 (720)	84.9 (720)		82.3 (413)	82.5 (414)	
Poorer	8.7 (74)	8.7 (74)	1.000	10.6 (53)	10.6 (53)	0.993
Middle	5.5 (47)	5.5 (47)		6.0 (30)	6.0 (30)	
Rich and richest	0.8 (7)	0.8 (7)		1.0 (6)	1.0 (5)	
**Marital status, % (** ***n*** **)**						
Ever married	24.6 (209)	11.0 (93)	0.000	60.8 (305)	23.1 (116)	0.000
Never married	75.4 (639)	89.0 (755)		39.2 (197)	76.9 (386)	
**Religion, % (** ***n*** **)**						
Muslim	98.4 (834)	98.4 (834)		98.6 (495)	98.4 (494)	
Non-muslim	1.7 (14)	1.7 (14)	1.000	1.4 (7)	1.6 (8)	0.606
**Earning status, % (** ***n*** **)**						
Non earning	99.4 (843)	43.8 (371)	0.000	98.6 (495)	29.3 (147)	0.000
Earning	0.6 (5)	56.2 (477)		1.4 (7)	70.7 (355)	
**Age at first marriage**						
Median age at first marriage (in years) (range, SD)	16 (15–19, 0.99)	17 (15–19, 1.03)	0.682	18 (15–22, 1.50)	18 (16–21, 1.03)	0.021

### Effect of Migration on Marital Status and age at Marriage

Migration was protective against early and child marriage. Urban migrant adolescents aged 15–19 years were 53% less likely to get married compared to their rural counterparts (aOR, 0.47; 95% CI, 0.34–0.66) (Table 2). In the 20–24 years age group, the urban migrants were 81% less likely to get married compared to their rural non-migrant counterparts (aOR, 0.19; 95% CI, 0.13–0.29). Girls earning an income had a significantly lower likelihood of getting married compared to their non-earning counterparts (aOR, 0.63; 95% CI, 0.41–0.97) in the 15–19 years age group (Table 2). Girls from to the middle wealth quintile group were at 2.19 (95% CI, 1.23–3.89) times higher risk of being married compared to the poorest category. No significant difference was noticed for other categories of wealth quintile.

**Table 2:** Effect of migration on marital status, age at first marriage and child marriage.

	**Marital status**		**Age at marriage**		**Child marriage**
**Characteristics**	**aOR (95% CI)**		**Coefficient (95% CI)**		**aOR (95% CI)**
	**Age 15–19**	**Age 20–24**	**Age 15–19**	**Age 20–24**	**Age 20–24**
**Migration**					
Rural control (ref.)
Rural to urban slum migrants	0.47^*^ (0.34, 0.66)	0.19^*^ (0.13, 0.29)	1.77^*^ (1.07, 2.46)	2.87^*^ (2.18, 3.55)	0.29^*^ (0.12, 0.69)
**Household wealth**					
Poorest (ref.)
Poorer	1.25 (0.82, 1.91)	1.26 (0.81, 1.96)	0.21 (−0.26–0.67)	0.12 (−0.42, 0.66)	1.02 (0.52, 1.99)
Middle	0.92 (0.52, 1.62)	2.19^*^ (1.23, 3.89)	−0.27 (−0.92, 0.37)	−0.47 (−1.10, 0.15)	0.99 (0.43, 2.25)
Rich and richest	1.82 (0.55, 6.00)	1.18 (0.32, 4.31)	−0.80 (−2.1, 0.50)	1.12 (0.43, 2.69)	0.56 (0.068, 5.19)
**Religion**					
Muslim (ref.)
Non–muslim	0.36 (0.08, 1.55)	2.16 (0.68, 6.89)	0.61 (−1.16, 2.38)	0.12 (−1.13, 1.36)	0.69 (0.13, 3.52)
**Earning status**					
Non-earning (ref.)
Earning	0.63^*^ (0.41, 0.97)	0.98 (0.63, 1.51)	−0.13 (−0.51, 0.26)	−0.05 (−0.48, 0.36)	0.93 (0.35, 2.42)

* *p < 0.05*.

Urban migrants had significantly higher age at marriage than the rural non-migrants for both 15–19 years age group (Coefficient, 1.77; 95% CI, 1.07–2.46) and 20–24 years age group (Coefficient, 2.87; 95% CI, 2.18–3.55) (Table 2).

### Effect of Migration on Child Marriage

Rural to urban migration was found protective against CM among women aged 20–24 years. Female rural-to-urban migrants in 20–24 age group were 71% less likely to get married before the age of 18 years compared to their rural counterparts (aOR, 0.29; 95% CI, 0.12–0.69) (Table 2).

## Discussion

Our study shows that rural to urban migrant adolescent girls (15–19 years) and young women (20–24 years) are less likely to be married compared to their rural counterparts. Additionally, age at first marriage of the among the urban migrants were higher than the rural non-migrants. The urban migrants aged 20–24 years were less likely to experience child marriage than the rural non-migrants. The existing literature from Bangladesh (14) and outside (18) support these findings. To our knowledge, this is the first-ever study which used propensity score matched cohort data for identifying the effect of migration on age at marriage and CM. The impact of migration on marriage using mixed research methods have been analyzed previously (14). However, the quantitative results were based on descriptive analyses only, where the control comprised of a sample from rural areas. The current paper used a propensity score matched sample, which minimized any biases in selecting the comparison group. This paper can be considered as a kind of extension of the paper by Naved (14) with more robust findings.

Although not part of the main results, some important predictors of CM need to be mentioned here. In contrast to the finding in the existing literature (15) we have found that among females aged 20–24, not the poor, but the households belonging to the middle wealth group were 19% more likely to marry off their daughters during childhood. A potential explanation may be that these households can quickly accumulate the resources required for arranging a wedding, including a dowry compared to the households in the economically disadvantaged group.

Question may arise as to how migration protects against child marriage. There is ample evidence that people migrate to urban areas for better opportunities and for better livelihoods. These opportunities may influence marital status, age at marriage, and also child marriage. Close-knit communities in rural Bangladesh and constant monitoring and supervision of adolescent girls' sexuality and control over their freedom (37) does not leave much room for negotiating delayed marriage by the girls' or their families. Ties with the community are relatively loose in urban slums of Dhaka. Exposure to a new environment and new opportunities influence the girls' world view. Economic and educational opportunities and higher exposure to media make them more confident and independent. They acquire bargaining power through economic and educational opportunities. As pointed out by Naved (14) engagement with income-earning may enable the girls to voice their opinion regarding timing of marriage and to defend that opinion while marriage decisions are made. On the other hand, the girls' families may be more relaxed about rushing their daughter's marriage as they depend on the financial support they get from earning daughters.

The findings from this paper and the existing literature seem to suggest that an income-earning migrant adolescent girl may be considered as an economic resource by the family rather than a burden to pass on through marriage (38). Migrant girls also are equipped with empowerment and knowledge (20) and this may allow them to have more autonomy, confidence and have higher opportunity to learn new knowledge and skills, which may allow them to explore new life perspectives (39). Although traditionally girls do not have any say in their own marriage (40) it is possible that as they are resourceful, they gain greater decision making power regarding timing of their own marriage (41), which enables delayed marriage and reduced CM.

### Strengths and Limitations

MHDSS and UHDSS are authentic and reliable sources of cohort data, which have been used in this paper. Collecting accurate data on age is challenging in Bangladesh since traditionally age is not tracked here and birth registration is a relatively recent phenomenon here, which started in 2001. This study benefitted from accurate age reporting in MHDSS, where information on birth was still being collected monthly when the non-migrant participants were born. Although data on age were carefully collected in the UHDSS, this surveillance was established recently and thus, the data cannot claim to be as authentic as MHDSS data.

The study had some other limitations as well. Some data (e.g., education, wealth, etc.) came from censuses conducted in MHDSS-2014 and UHDSS-2016 at different time points. As education, for instance, may change over time for the adolescent girls, we were unable to use information on education PSM though it is an important confounder in measuring the effect on age at marriage (42). Other potential confounders could not be adjusted due to unavailability of data. Due to some issues around access, we were unable to take advantage of the longitudinal nature of the data, which made us use analytical methods appropriate for cross-sectional data. Despite this limitation, use of longitudinal data ensured us greater accuracy of data due to shorter recall periods.

The residents of Dhaka slums actually come from different parts of Bangladesh and not only from Matlab. In that sense, the control in this study may not be the perfect match for the urban migrants. However, using PSM we were successful in deriving a sample, where the migrants were comparable to the sample from Matlab by some background characteristics.

The findings are only applicable for adolescent migrants living in urban slums of Dhaka and are not generalizable for all urban population.

Despite these limitations, this study presents valid findings on effect of rural to urban migration on age at marriage and CM in Bangladesh.

## Conclusion

Findings from this study clearly demonstrate that CM in Bangladesh can be reduced not only by increased education, but also by creating economic opportunities for girls.

## Data Availability Statement

The original contributions presented in the study are included in the article/supplementary materials, further inquiries can be directed to the corresponding author/s.

## Author Contributions

JA, KP, MM, and RN were involved in the conception and design of conceptualization, findings interpretation, and review and finalization of the paper. JA and HS were involved in data analyses, interpretation, and review and finalization of the findings. All authors have given final approval of the version to be published.

## Funding

Swedish International Development Cooperation Agency (SIDA) funded this work. The funder provided support in the form of salaries for authors but did not have any additional role in the study design, analysis, decision to publish, or preparation of the manuscript.

## Conflict of Interest

The authors declare that the research was conducted in the absence of any commercial or financial relationships that could be construed as a potential conflict of interest.

## Publisher's Note

All claims expressed in this article are solely those of the authors and do not necessarily represent those of their affiliated organizations, or those of the publisher, the editors and the reviewers. Any product that may be evaluated in this article, or claim that may be made by its manufacturer, is not guaranteed or endorsed by the publisher.
